# Extraordinary increase in West Nile virus cases and first confirmed human Usutu virus infection in Hungary, 2018

**DOI:** 10.2807/1560-7917.ES.2019.24.28.1900038

**Published:** 2019-07-11

**Authors:** Anna Nagy, Eszter Mezei, Orsolya Nagy, Tamás Bakonyi, Nikolett Csonka, Magdolna Kaposi, Anita Koroknai, Katalin Szomor, Zita Rigó, Zsuzsanna Molnár, Ágnes Dánielisz, Mária Takács

**Affiliations:** 1 National Reference Laboratory for Viral Zoonoses; National Public Health Center, Budapest, Hungary; 2 These authors contributed equally to this work; 3 Department of Communicable Diseases Epidemiology and Infection Control; National Public Health Center, Budapest, Hungary; 4 Institute of Medical Microbiology, Semmelweis University, Budapest, Hungary; 5 Department of Microbiology and Infectious Diseases, University of Veterinary Medicine, Budapest, Hungary; 6 Viral Zoonoses, Emerging and Vector-borne Infections Group, Institute of Virology, University of Veterinary Medicine, Vienna, Austria; 7 National Reference Laboratory for Viral Exanthematous Diseases; National Public Health Center, Budapest, Hungary

**Keywords:** West Nile virus, Hungary, Usutu virus, neuroinvasive disease, human infection

## Abstract

**Background:**

During the 2018 WNV transmission season, similarly to other endemic areas in Europe, a large number of human West Nile virus (WNV) infections were reported in Hungary.

**Aims:**

We summarise the epidemiological and laboratory findings of the 2018 transmission season and expand experiences in flavivirus differential diagnostics.

**Methods:**

Every patient with clinical suspicion of acute WNV infection was in parallel tested for WNV, tick-borne encephalitis virus and Usutu virus (USUV) by serological methods. Sera, whole blood and urine samples were also tested for the presence of viral nucleic acid.

**Results:**

Until the end of December 2018, 215 locally acquired and 10 imported human WNV infections were notified in Hungary. All reported cases were symptomatic; most of them exhibited neurological symptoms. In a large proportion of tested individuals, whole blood was the most appropriate sample type for viral nucleic acid detection, but because whole blood samples were not always available, testing of urine samples also extended diagnostic possibilities. In addition, the first human USUV infection was confirmed in 2018 in a patient with aseptic meningitis. Serological cross-reactions with WNV in different serological assays were experienced, but subsequent molecular biological testing and sequence analysis identified Europe lineage 2 USUV infection.

**Conclusion:**

Careful interpretation and simultaneous application of different laboratory methods are necessary to avoid misdiagnosis of human USUV cases. Expansion of the laboratory-confirmed case definition criteria for detection of viral RNA in any clinical specimens to include urine samples could increase diagnostic sensitivity.

## Background

West Nile virus (WNV) and Usutu virus (USUV) are phylogenetically closely related mosquito-borne members of the family *Flaviviridae*, and belong to the Japanese encephalitis antigenic complex of the *Flavivirus* genus [1,2]. Both viruses have been isolated from numerous ornithophilic mosquito species, mainly *Culex* spp. [1,2]. In the enzootic cycle of WNV and USUV, avian species are also involved and serve as amplifying hosts. Mosquitoes facilitate virus transmission to humans and equids which then remain incidental hosts as they are not able to produce a level of viraemia sufficient for further virus transmission by mosquito bites [2]. The possibility of WNV and/or USUV transmission via blood transfusion or organ transplantation has also been described, raising awareness of the need for preventive measures ensuring blood safety during the transmission period, usually between July and November [3-7]. However, additional transmission routes of human WNV infection including occupational transmission in laboratory settings, transplacental transmission and transmission via breast milk or during delivery have rarely been described [8-11].

According to the seasonal WNV surveillance data of the European Centre for Disease Control and Prevention (ECDC), an overall 2.8-fold increase in the number of reported human WNV cases was observed in the European Union (EU) in 2018 (n = 1,503) compared with the total number of cases from the previous three years (n = 537) [12]. 

Because clinically manifest cases of human USUV infections are rarely detected, only limited knowledge is available about the clinical relevance of USUV infections in humans. In 2009, two USUV-related human neuroinvasive infections were reported in immunocompromised individuals in Italy [13,14]. In 2013, three other neurological infections were reported by Croatia [15] and an idiopathic facial paralysis was associated to USUV infection in France in 2016 [16]. Furthermore, USUV circulation or co-circulation with WNV in certain EU countries has been confirmed by screening healthy blood donors. Currently available WNV RNA detection systems, used for testing blood supplies, show cross-reactivity to USUV. Therefore, USUV nucleic acid was detected on several occasions in the past 3 years, for example in Austria, Germany and Italy [6,7,17,18].

In Hungary, human neuroinvasive WNV infections were first documented in 2003 [19], while USUV was until recently only detected in animal specimens [20,21]. Since 2003, West Nile fever (WNF) and the more severe manifestation of the infection, West Nile neuroinvasive disease (WNND) have been regularly diagnosed in Hungary with average 15–20 cases annually [22]. Usutu virus was first detected in organ samples of a blackbird in 2005 [20] and since then, continuous circulation of the virus has been recorded in Hungary [21]; however, there has not been any laboratory evidence for human infections until now.

The aim of the study was to summarise the epidemiological and laboratory findings of the 2018 WNV transmission season and to improve diagnostic experiences by comparing the possibility of viral nucleic acid detection in different sample types used for molecular diagnostics. We also draw attention to the challenges of the laboratory diagnostics of human flavivirus infections; a detailed case description of the first human USUV infection provides strong evidence for the need of comprehensive diagnostic approach in areas were two or more human pathogenic flaviviruses are co-circulating.

## Methods

### Epidemiological surveillance and data collection

Until 2008, WNV infections had been reported in Hungary only within the syndromic surveillance system, as aseptic meningitis and/or infectious encephalitis. Since then, WNV infections have become notifiable by law. The European Union’s (EU) clinical and epidemiological case definition criteria [23] are applied for both confirmed and probable WNV infections.

The comprehensive and nation-wide operation of the Hungarian surveillance system for WNV is compulsory by law. All physicians, including general practitioners, specialists working in outpatient clinics, physicians working in hospitals and at emergency services, pathologists, and microbiologists should report all clinically suspected or confirmed WNV infections. To avoid case duplications, all information from different sources is finally collected and reported by the Department of Communicable Diseases Epidemiology and Infection Control; National Public Health Center (NPHC). 

In Hungary, there is a central national database, where online connection with data suppliers, such as physicians and other specialists is available. All data on WNV infections and microbiological laboratory results are entered into the database directly. The password-protected web-based system is also accessible for the local, county and national public health authorities (district and county governmental offices, National Public Health Centre).

The Department of Communicable Diseases Epidemiology and Infection Control at the National Public Health Centre in Budapest, Hungary collects data on demographic characteristics, clinical manifestations, date and duration of hospitalisation, outcome of illness (full recovery, recovery with sequelae or death) and laboratory results of all reported WNV infections. In 2012, standardised epidemiological investigation forms were introduced to obtain a detailed travel history during the incubation period (2–14 days before symptom onset) and to identify the suspected place of exposure. The Department of Communicable Diseases Epidemiology and Infection Control at the NPHC reports the epidemiological and microbiological data directly to The European Surveillance System (TESSy) operated by the ECDC.

### Public health implications and response actions

In accordance with the European Commission Directive 2014/110/EU [24], Hungary currently applies a 28-day deferral of prospective blood donors who have visited or live in an affected area, as a preventive measure to avoid transfusion-transmitted WNV infections. Immediate notification of WNF cases to the Hungarian National Blood Transfusion Service is accomplished by the Department of Communicable Diseases Epidemiology and Infection Control at the NPHC.

Awareness-raising campaigns for primary prevention of insect bites are also implemented, including preparation of documents which are available on the NPHC’s website to provide comprehensive information to the public.

Owing to the weather conditions in the early spring of 2018, characterised by precipitation anomalies, a nationwide public health mosquito control started in April and took place in 977 settlements of Hungary.

### Laboratory case definitions and diagnostic algorithm

WNV infections are defined as laboratory-confirmed or probable cases according to the EU case definition criteria [23]. For laboratory case confirmation, at least one of the following four criteria must be met: (i) isolation of the virus from blood or cerebrospinal fluid (CSF), (ii) detection of WNV nucleic acid in blood or CSF, (iii) detection of WNV-specific IgM antibodies in CSF or (iv) detection in serum of anti-WNV IgM antibodies at high titre and detection of anti-WNV IgG antibodies and confirmation by neutralisation. The presence of WNV-specific antibodies in a serum sample allows only probable case classification. Laboratory results need to be interpreted according to flavivirus vaccination status.

The laboratory diagnosis of human viral zoonotic infections across the entire territory of Hungary is centralised and performed only at the National Reference Laboratory (NRL) for Viral Zoonoses of the NPHC. Microbiological results of laboratory-confirmed or probable WNV infections are directly reported to the Department of Communicable Diseases Epidemiology and Infection Control at the NPHC. Serological differential diagnostic tests for tick-borne encephalitis virus (TBEV) and WNV are routinely performed in all serum and CSF samples sent to the laboratory with the clinical suspicion of aseptic meningitis and/or infectious encephalitis. Since the beginning of the 2018 WNV transmission season, routine testing for USUV has also been introduced. Besides the serological investigation, molecular diagnostic assays are also applied for WNV and USUV because of the close serological relatedness of the two viruses. Furthermore, for human WNV cases, the diagnostic algorithm also covers lineage determination by Sanger sequencing; the sequencing results are regularly reported to the ECDC. 

The Hungarian NRL for Viral Zoonoses is in close cooperation with the Hungarian NRL for Viral Exanthematous Diseases at the NPHC. The standard panel for investigating the aetiology of exanthematous illnesses includes screening for measles, rubella and/or parvovirus B19 viruses, while testing for WNV is in many cases not requested by the clinicians. As a result of the collaboration between the two reference laboratories, samples obtained from patients with exanthema, fever, myalgia and/or arthralgia are also involved in WNV screening during the transmission period.

## Results

### Epidemiology of human West Nile virus infections in Hungary in 2018

**Figure 1:**
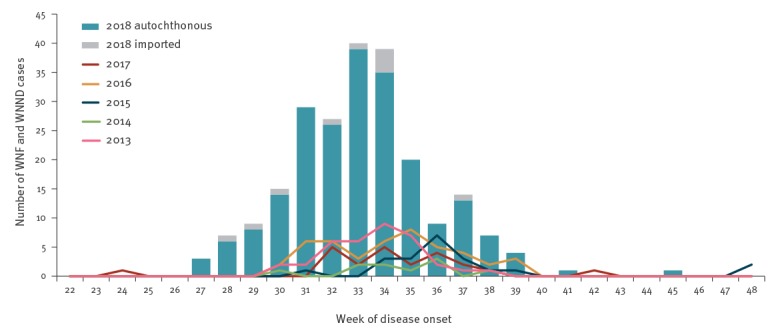
Number of autochthonous and imported human cases of West Nile virus infection by week of symptom onset, Hungary, 2018 (n = 225), compared with 2013–2017 (n = 139)

WNF: West Nile fever; WNND: West Nile neuroinvasive disease.

In 2018, the first locally acquired human WNV infection was diagnosed during week 28 (week starting 9 July 2018), with symptom onset in week 27 (week starting 2 July 2018), much earlier than generally in previous years (Figure 1). In 2016 and 2017, for instance, reporting started in weeks 33 and 34, respectively (weeks starting 15 August 2016 and 21 2017). The earliest week of disease onset occurred in 2017, with one probable case who developed clinical signs of WNV infection during week 24 (week starting 12 June 2017) (Figure 1). 

The Figure presents all cases who met the criteria for laboratory-confirmed and probable cases. In 2017, symptom onset was dated back to a probable case of infection in week 24, for whom laboratory investigation was initiated during week 27.

Based on date of symptom onset, altogether 215 autochthonous laboratory-confirmed and probable WNV infections were reported in 2018 between weeks 27 and 45, with a peak in August (between weeks 31 and 35, n = 141; 62.6%) (Figure 1). Based on travel history obtained, an additional 10 imported infections were recorded; hence the overall number of laboratory-diagnosed human WNV cases was 225. Imported cases were related to the following countries: Austria (one confirmed case), Belgium (one probable case), Croatia (two confirmed cases), Romania (two confirmed cases), Serbia (one confirmed case, two probable cases) and Turkey (one confirmed case). 

**Figure 2:**
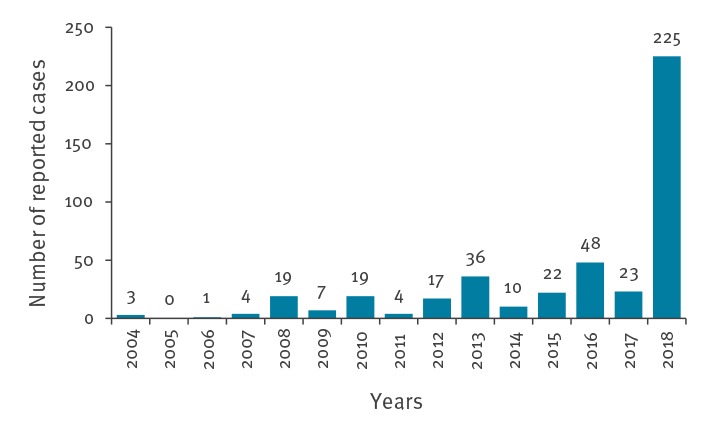
Number of reported autochthonous and imported human cases of West Nile virus infection by year, Hungary, 2004–2018 (n = 438)

The cumulative number of cases in 2018 represents a more than ninefold increase compared with the 2017 transmission period (n = 23) (Figure 2). Figure 2 presents the number of reported human WNV infections of Hungary by year, showing that the number of autochthonous and imported cases in 2018 (n = 225) exceeded the cumulative sum of cases in the previous 14 years (n = 213).

Reporting of human West Nile virus cases started in 2004, therefore epidemiological data are summarised between 2004 and 2018. The figure shows both probable and confirmed cases.

**Figure 3:**
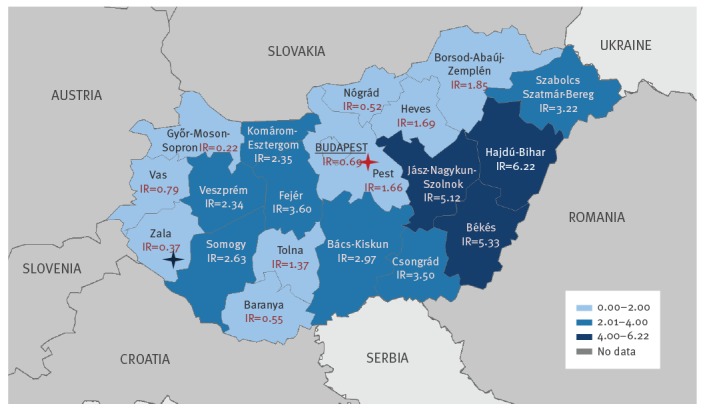
Geographical distribution of laboratory-diagnosed cases of West Nile fever and West Nile neuroinvasive disease by NUTS 3 region, Hungary, 2018 (n = 215)

IR: incidence rate; NUTS: Nomenclature of Territorial Units for Statistics; WNV: West Nile virus.

The geographical distribution of the laboratory-diagnosed human WNV infections at NUTS 3 (Nomenclature of Territorial Units for Statistics 3) level, shows that WNV was circulating in the entire country (Figure 3). The highest incidence of WNV infections was estimated in the eastern part of Hungary, which is highly affected every year. In 2018, for the first time, a human WNV infection was diagnosed in Zala county, Western Hungary (Figure 3). The national incidence rate (WNV cases/100,000 inhabitants) in Hungary varies within a range of 0.01 (2006) and 0.5 (2016) annually (data not shown). By the end of the 2018 transmission season, an incidence rate of 2.3 was measured, which indicates more than a 4.5-fold increase compared with the previous transmission period.

The incidence rates refer to WNV infections/100,000 inhabitants. Blue star: county where a human WNV infection was reported for the first time. Red star: residence of the first confirmed human Usutu virus case.

The median age of patients was 57 years (IQR (interquartile range): 22 years). The proportion of male cases was 56.9% (n = 128). Most of the patients (70.2%; n = 158) developed symptoms corresponding to WNND, while 28.9% of infections (n = 65) could be characterised as WNF. Information on the clinical symptoms of two patients (< 1%) is currently missing or incomplete. Exanthematous symptoms such as a morbilliform rash were observed in 21.8% of cases (n = 49): 11 patients (4.9%) developed maculopapular exanthema together with neurological symptoms, while the remaining 38 (16.9) patients met the clinical course of classical WNF. The mean duration of hospitalisation was 9.1 days (IQR: 8 days). Fifteen cases were fatal.

### Virological laboratory results

According to the EU laboratory case definition criteria [23], 138 confirmed and 87 probable WNV infections were reported in 2018. Seroconversion or a titre increase of at least fourfold could be observed in 23.2% (n = 32) of confirmed cases. The CSF sample was positive for WNV-specific IgM antibodies in 47.8% (n = 66) of patients, while case confirmation was based on viral nucleic acid detection in blood in 29.0% (n = 40) of patients. WNV-specific IgG, IgA and IgM antibody detection in serum and CSF was performed for all cases, while viral nucleic acid detection was carried out for 73.8% (n = 166) of patients. EDTA-treated whole blood and urine samples, the most appropriate specimens for viral RNA detection, were available only from 47.6% (n=107) and 63.6% (n = 143) of patients, respectively. In total, 53 patients (31.9%) were positive for WNV RNA.

Detailed results of the distribution of viral RNA positivity in different sample types are summarised in Table 1.

**Table 1:** Distribution of PCR-positive results for West Nile virus by sample type and patient, Hungary, 2018 (n = 294)

Nucleic acid detection by sample type	Number of tested samples	Number of PCR-positive samples
EDTA-treated whole blood	107	39
Serum	44	6
Urine	143	35
**PCR result by sample type**	**Number of PCR-positive patients**	**Percentage among all PCR-positive patients (n = 53)**
Whole blood PCR− and serum PCR+	1	1.9%
Whole blood PCR+ and urine PCR+	24	45.3%
Whole blood PCR+ and urine PCR−	10	18.9%
Whole blood PCR− and urine PCR+	3	5.7%
Whole blood PCR+ and urine not available	5	9.4%
Urine PCR+ and whole blood not available	8	15.1%
Only serum sample available PCR+	2	3.8%

+: positive; −: negative; EDTA: ethylenediaminetetraacetic acid.

Partial sequencing of the NS3 protein-coding region of the WNV genome could be done for 49 cases (GenBank accession numbers for 34 patients for whom sequencing could be carried out for both strands: MK224611–MK224644). Similarly, to previous years, WNV lineage 2 was identified in all of them [25]. 

From the beginning of July to the end of September 2018, clinical specimens from altogether 69 exanthematous patients were examined by the Hungarian NRL for Viral Zoonoses in collaboration with the Hungarian NRL for Viral Exanthematous Diseases. In accordance with previous years’ experiences [26], some of these patients (n = 23) were reported to the laboratory as clinically suspected measles virus infections, hence investigation for WNV was mostly conducted regardless of the clinicians’ request. Neither measles nor rubella virus infections could be confirmed during the given time period; however, in 23 of the 69 patients (33.3%), WNV infections were reported. Differential diagnostic tests for other endemic flaviviruses such as TBEV and USUV were also performed to exclude possible serological cross-reactions. In eight patients (5.1% of WNND cases), clinicians requested only laboratory investigation for TBEV, while acute WNV infection was confirmed by serological testing that was done in parallel.

### Case description of an autochthonous Usutu virus infection

**Figure 4:**
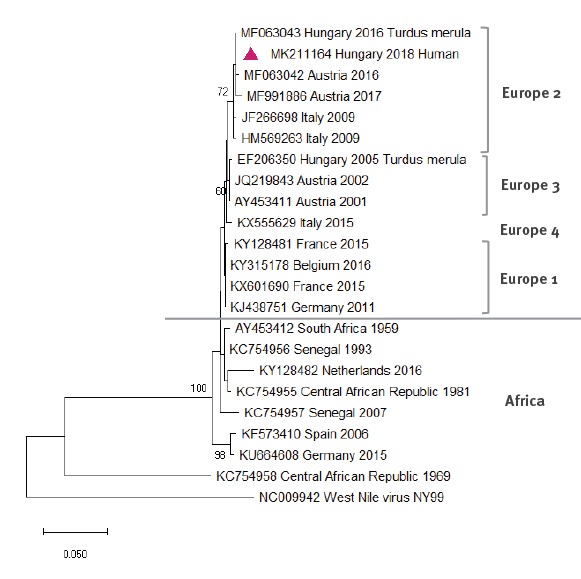
Phylogenetic tree of Usutu virus sequences based on alignments of the NS5 partial region of the genome compared with patient sequence, Hungary, 2018

USUV: Usutu virus; WNV: West Nile virus.

All whole blood and urine samples that gave negative results in the WNV RT-qPCR assay were further tested for the presence of USUV. In one whole blood sample from 2018, USUV RNA was detected. Therefore, this patient was classified as a confirmed case of USUV infection, after having first been interpreted as a probable WNV infection based only on WNV serological results. A possible co-infection with WNV was excluded by subsequent molecular testing and by comparing IgM, IgA and IgG antibody end-point titres for USUV and WNV. A considerable titre difference between USUV- and WNV-specific antibodies, together with positive USUV and negative WNV results in PCR confirmed an ongoing USUV infection (Table 2). Based on the partial sequence of the NS5 region, obtained in the nested PCR from the blood sample, USUV lineage Europe 2 was identified (Figure 4). The 391 nt long sequence of the USUV NS5 gene obtained from the human clinical sample (GenBank accession number: MK211164) showed 100% identity with a strain (GenBank accession number: MF063043), that was detected in a blackbird in 2016 (Figure 4). 

**Table 2:** Summary of laboratory results of confirmed human Usutu virus infection, Hungary, 2018

Serum sample analysis					
Immunofluorescence assay					
Anti-USUV IgG	1:160	Anti-WNV IgG	1:20	Anti-TBEV IgG	1:10
Anti-USUV IgM	1:640	Anti-WNV IgM	1:40	Anti-TBEV IgM	< 1:10; negative
Anti-USUV IgA	≥ 1:1,280	Anti-WNV IgA	1:20	Anti-TBEV IgA	< 1:10; negative
ELISA					
WNV IgM capture ELISA: index value = 4.75; positive					
Molecular diagnostic results		EDTA-treated whole blood		Urine	
WNV RT-qPCR		Negative		Negative	
USUV RT-qPCR		Positive Ct 37.00		Negative	
USUV nested RT-PCR		Positive		Negative	

EDTA: ethylenediaminetetraacetic acid; ELISA: enzyme-linked immunosorbent assay; TBEV: tick-borne encephalitis virus; USUV: Usutu virus; WNV: West Nile virus.

We used in-house developed indirect immunofluorescent assays for IgG, IgM and IgA detection of USUV, WNV and TBEV [26], a commercial ELISA (Focus Diagnostics, DiaSorin Molecular LLC, Cypress, United States) and an in-house developed quantitative RT-PCR [34]. Cut-off for antibody titres: <1:10: negative; ≥1:10: positive; 1:10: indeterminate. All samples were taken 10 days after symptom onset.

Phylogenetic reconstruction was performed by MEGA-X software using the neighbour-joining method based on the Kimura two-parameter model with 1,000 bootstrap replicates. Values on the branches represent the percentage of bootstrap replicates. The sample obtained from the Hungarian patient is indicated by a red triangle (GenBank accession number: MK211164). USUV strains were named as follows: GenBank accession number, country of origin and year of detection or isolation. WNV was used as outgroup to root the tree.

The USUV patient who presented with symptoms of aseptic meningitis was a man in his 40s residing in Pest County, Central Hungary (Figure 3), with no travel history at least 2 weeks before the onset of disease. Symptoms started on 8 September 2018; blood and urine samples were taken and sent to the diagnostic laboratory 10 days later, on 18 September. The course of the disease was characterised by symptoms such as muscle spasm, chills, fever, headache and nuchal rigidity. The duration of hospitalisation was 7 days. The patient, who had no known immunosuppression or other co-morbidities recovered without neurological sequelae.

## Discussion

During the 2018 transmission season, the largest number of human WNV infections since 2003 was reported in Hungary, 215 autochthonous and 10 imported cases. The total number of cases reported in that year exceeded the cumulative total of cases of the previous 14 years. The early start of the WNV season and a significantly larger number of cases has also been noted across Europe [27]. Currently, the most likely explanation for this trend is that the weather conditions, elevated temperature and precipitation anomalies, and potentially other environmental factors were favourable for the early expansion of the vector population [5,27]. 

In Hungary, WNV infections were present throughout the entire country, with higher incidence in the East. There are no considerable differences in the proportion of the elderly population between eastern and western regions. However, in Hajdú-Bihar county, the region where the highest incidence rate (IR = 6.22) was calculated there is a regional central hospital and a university teaching hospital, which both have infectious diseases and general neurology wards. The availability of medical care services and the increased interest of local clinicians can be a possible explanation for the elevated number of cases. Most of the reported WNV cases (70.2%) exhibited neurological involvement, even though milder or asymptomatic forms of the infection might be more frequent. Patients with severe clinical symptoms are more likely to seek medical care, whereas asymptomatic WNV infections and patients with mild WNF are less frequently detected, leading to the over-representation of laboratory confirmed WNND cases.

Besides locally acquired infections, a number of cases were imported from other WNV-endemic countries such as Austria, Croatia, Romania, Serbia and Turkey. In addition, a probable WNF was imported from Belgium. The patient, who lives in Belgium, started to present clinical signs of acute viral infection 1 day after arriving to Hungary for vacation. Although viral nucleic acid detection was negative, anti-WNV IgG antibody titre was fourfold higher than anti-USUV IgG level; this titre difference was high enough to be indicative of WNV infection. Furthermore, the patient was negative for anti-USUV IgM or IgA antibodies and positive for anti-WNV IgM and IgA. Therefore, in this case an acute WNV infection could be supposed, even though to the best of our knowledge Belgium has not reported any human cases.

Based on our sequence data, lineage 2 WNV strains were identified as aetiological agents of the human infections, correlating with both human and animal data of previous years [28-30]. Lineage 2 WNV strains are predominant in the Eastern Mediterranean and Central European region, including Hungary.

Compared with WNV, USUV is less likely to be associated with severe infections in humans, except in immunocompromised individuals [13,14]. Only a few clinically manifested cases have been described so far [15,16,31]. Here we report the first human USUV infection in Hungary; it had a neurological manifestation, characterised as aseptic meningitis. Based on the 391 nt fragment of the NS5 region, USUV lineage Europe 2 was identified, with 100% identity to the HU-47540/16 strain that was detected in a blackbird in the western part of Hungary in 2016 [21]. Very similar lineage Europe 2 USUV strains are also circulating in Lower Austria [21]. 

WNV outbreaks among equids were also regularly reported to the ECDC by the Hungarian National Food Chain Safety Office which is responsible for the laboratory diagnosis and reporting of animal cases of WNV infections. A passive surveillance system of birds is also maintained by the Hungarian National Food Chain Safety Office for WNV and USUV. 

Human USUV infection has not been laboratory-confirmed in Hungary so far, this is the first locally acquired human case with clinical manifestation. In the case of this patient, additional molecular examination of the whole blood sample revealed an acute USUV infection. Serological cross-reactions with WNV were demonstrated in both immunofluorescence and ELISA tests. Cross-reactivity is often experienced not only in serological tests but also in molecular diagnostic assays, which may create further diagnostic challenges [6,7]. Therefore, laboratory results need to be carefully interpreted, especially in countries like Hungary where at least three human pathogenic flaviviruses (WNV, USUV and TBEV) are circulating. Parallel laboratory testing should always be conducted to exclude serological cross-reactions and to avoid misdiagnosis of possible secondary flavivirus infections or co-infections. 

Owing to the highly elevated number of clinical samples, confirmation by virus neutralisation assay was not an integrative part of the daily laboratory routines in 2018; however, by the end of the transmission season, samples in which both elevated anti-USUV and anti-WNV IgG immunofluorescence titres have been detected were also confirmed by using neutralisation assay.

According to the literature, the use of either urine or whole blood samples for molecular diagnostics instead of serum improved the efficiency of viral RNA detection [32,33]; however, the appropriate sample types were available for only half of the patients. In addition, the current case definition criteria in the EU specify ‘Detection of WNV nucleic acid in blood or CSF’ as laboratory test for case confirmation of a WNV infection. In our studies, urine was positive for WNV by PCR in 5.7% of cases, while whole blood was negative for these cases. In 15.1% of patients, urine was PCR-positive for WNV, while the blood sample collected into EDTA-treated tubes were not sent to the laboratory, therefore whole-blood PCR results were not available. Modification of the case definition by including isolation of the virus or detection of WNV nucleic acid in any samples could increase diagnostic sensitivity.

The known geographic distribution of USUV is wider than the distribution of WNV in Europe. Molecular testing of healthy people (blood donors) in Germany and Austria revealed that human USUV infections may be more frequent than WNV infections [5-7]. The current report emphasises the neuroinvasive potential of USUV in a human patient without known immunosuppression or comorbidities. Targeted investigations of neurological cases in other USUV-affected countries might reveal USUV in the aetiology of further human cases. These findings and recently published evidence of USUV and WNV co-infection [5] underline the importance of thoroughly performed serological tests combined with molecular assays to differentiate WNV and USUV infections and to avoid unrecognised human USUV cases.
